# KLF8 promotes tumorigenesis, invasion and metastasis of colorectal cancer cells by transcriptional activation of FHL2

**DOI:** 10.18632/oncotarget.4517

**Published:** 2015-07-15

**Authors:** Qingqing Yan, Wenjing Zhang, Yao Wu, Meiyan Wu, Mengnan Zhang, Xinpeng Shi, Jinjun Zhao, Qingzhen Nan, Ye Chen, Long Wang, Tianming Cheng, Jiachu Li, Yang Bai, Side Liu, Jide Wang

**Affiliations:** ^1^ Guangdong Provincial Key Laboratory of Gastroenterology, Department of Gastroenterology, Nanfang Hospital, Southern Medical University, Guangzhou, China; ^2^ Department of Rheumatism, Nanfang Hospital, Southern Medical University, Guangzhou, China; ^3^ Division of Vascular Interventional Radiology, Third Affiliated Hospital, Sun Yat-sen University, Guangzhou, China; ^4^ Department of Oncology, The First Affiliated Hospital of Chongqing Medical University, China

**Keywords:** KLF8, EMT, FHL2, metastasis, colorectal cancer

## Abstract

The transcription factor Krüppel-like factor (KLF)8 plays an important role in the formation of several human tumors, including colorectal cancer. We recently identified four-and-a-half LIM protein 2 (FHL2) as a critical inducer of the epithelial-to-mesenchymal transition (EMT) and invasion. However, the molecular mechanism by which KLF8 affects FHL2-mediated tumor proliferation, EMT and metastasis remains unknown. Here, we showed that KLF8 overexpression promoted EMT and metastatic phenotypes. KLF8 expression was stimulated by transforming growth factor (TGF)-β1. Moreover, KLF8 acted as a potential EMT inducer by stimulating vimentin expression and inducing a loss of E-cadherin in stable KLF8-transfected cells. KLF8 overexpression induced a strong increase in FHL2 expression, and a positive correlation between the expression patterns of KLF8 and FHL2 was observed in CRC cells. Promoter reporter and chromatin immunoprecipitation (ChIP) assays demonstrated that KLF8 directly bound to and activated the human FHL2 gene promoter. However, siRNA-mediated repression of *FHL2* in KLF8-overexpressing cells reversed the EMT and the proliferative and metastatic phenotypes. *In vivo*, KLF8 promoted FHL2-mediated proliferation and metastasis via orthotopic implantation. Taken together, this work identified KLF8-induced FHL2 activation as a novel and critical signaling mechanism underlying human breast/colorectal cancer invasion and metastasis.

## INTRODUCTION

Specificity protein 1 (Sp1) and other Sp and Krüppel-like factor (KLF) proteins are members of a family of transcription factors [1–2]. Sp1-Sp9 proteins regulate expression of multiple genes in normal tissues and tumours [1, 3]. Other proteins KLFs are essential DNA-binding transcriptional regulators with diverse functions in various cellular processes, including differentiation [4], proliferation [5], inflammation [6], migration [7], and pluripotency [8]. Recent progress has highlighted the significance of KLFs in tumor progression. For example, KLF family proteins have been shown to have either a tumor-promoting (KLF4 [2, 7], KLF6 [9], KLF9 [10], KLF10 [11], KLF11 [12], and KLF17 [13]) or a tumor-suppressing (KLF5 [14], KLF12 [15]) role by intervening in cell signaling associated with proliferation and/or suppression.

KLF8 [16] is a member of the KLF transcription factor family and binds to a DNA consensus sequence (GT-box [17]: 5′-CACCC-3′) to regulate transcription. KLF8 is ubiquitously expressed in proliferating and regenerating mammalian cells and is highly expressed in various types of human malignancies [18, 19]. Recent studies have suggested that KLF8 plays an important role in gastric cell development and progression [16]. For example, the downregulation of KLF8 expression inhibited hepatocellular carcinoma cell growth, migration, and invasion by decreasing cyclin D1, Cdk, matrix metalloproteinase (MMP)-2 and MMP-9 expression [17]. In addition, KLF8 induced tumor cell epithelial-to-mesenchymal transition (EMT) and maintained the invasive potential of cancer, which appeared to play a crucial role in the metastatic progression of human carcinomas [16].

Four-and-a-half LIM-only protein 2 (FHL2) [20], also known as downregulated in rhabdomyosarcoma LIM (DRAL) domain protein, is a member of the FHL family and comprises four LIM domains and one N-terminal half LIM domain. The LIM domains are double zinc finger motifs that play multiple roles in protein-protein interactions such as functional modifiers and adaptors [21, 22]. FHL2 is a LIM-only protein that participates in cell transcription and signal transduction. It functions as a co-activator of several transcription factors, such as β-catenin [23], AP-1 [24], CREB [25], and others [26]. We and others have implicated FHL2 as an oncogenic factor in colorectal cancer (CRC) [21], squamous cell carcinoma [27], melanoma [28], glioblastoma [29] and prostate cancer [30]. The over-expression of FHL2 in CRC cells is essential to maintain their malignant phenotype and might be a mediator or a trigger of EMT in CRC cells [23, 31]. We identified the positive correlation between Sp1 and FHL2 in expressing pattern of GI cancers [3]. However, the mechanism by which KLF8 regulates FHL2 expression through transcriptional activation to promote cell growth, EMT, invasion and metastasis has not been investigated.

In this study, we found that KLF8 overexpression promotes EMT and invasive and metastatic phenotypes. KLF8 regulated FHL2 expression through the GT-box located in the FHL2 proximal promoter. FHL2 short interfering RNA (siRNA) reversed these effects in cells that overexpressed KLF8. These results indicated that tumor progression and metastasis were promoted by a key signaling pathway involving KLF8-mediated regulation of FHL2. These findings further uncovered the role of KLF8 in CRC invasion and metastasis.

## RESULTS

### KLF8 upregulation promotes epithelial-mesenchymal transition (EMT)

**Figure 1:**
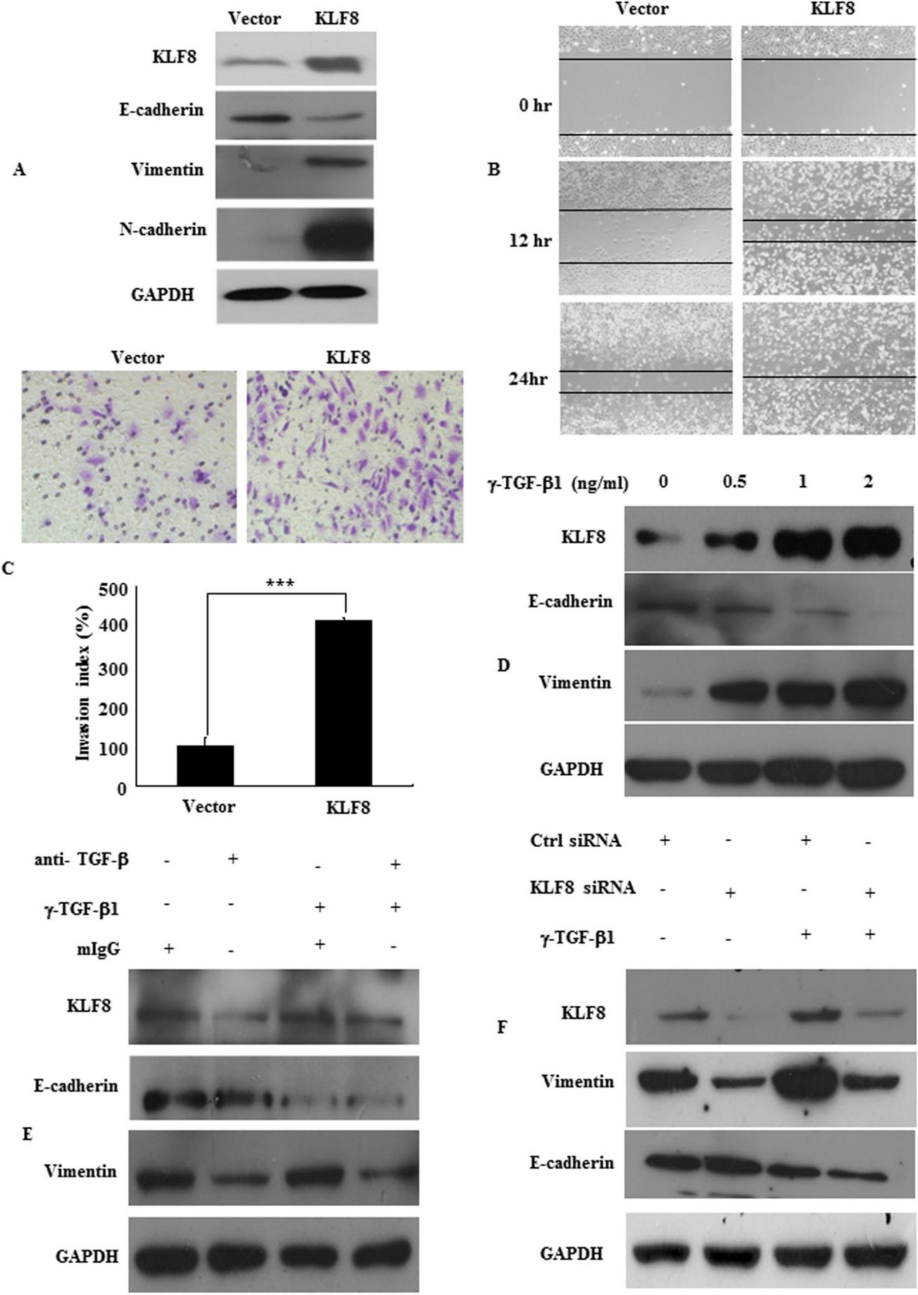
KLF8 upregulation promotes EMT **A.** KLF8, E-cadherin, vimentin, N-cadherin expression in stable transfectants in LoVo cells, detected by western blot. **B.** Images of the wound closure of monolayer LoVo stable transfectants. Original magnification, 10×. **C.** Invasive potential of LoVo stable transfectants transfected with the vector, ***, *p* < 0.001. **D.** KLF8 expression in LoVo cells treated with the indicated concentrations of recombinant γ-TGF-β1 for 48 h. The protein levels of KLF8, E-cadherin and vimentin were detected by western blot. **E.** LoVo cells were treated with recombinant TGF-β1 (γ-TGF-β1; 2 ng/ml) in the presence of neutralizing anti-TGF-β1 antibody (α-TGF-β, 2 μg/ml) or mouse IgG (mIgG) for 48 h. The expression of KLF8, E-cadherin and vimentin was detected by western blot. **F.** Stable KLF8 transfectants were seeded in 6-well plates overnight and treated with γ-TGF-β1 (2 ng/ml) for an additional 48 h. The expression of KLF8, E-cadherin and vimentin was detected by western blot. All figures are representative of four independent experiments with similar findings.

EMT is the key process that drives cancer metastasis [7, 13, 16, 23, 31–34]. The loss of E-cadherin expression and gain of vimentin expression are considered to be the most important molecular markers of EMT. Our previous reports demonstrated that KLF8 was overexpressed in CRC (Shi X, submitted). Here, we demonstrated that KLF8 overexpression promotes EMT in CRC in LoVo cells. KLF8 overexpression was confirmed by western blot analysis (Figure 1A). EMT induction was demonstrated by a shift in the expression of epithelial markers (E-cadherin) to mesenchymal markers (vimentin and N-cadherin; Figure 1A).

We also demonstrated increased migration and invasion. As assessed by a wound-healing assay, in which a gap is formed in a cell monolayer, KLF8 overexpression markedly accelerated cell migration (Figure 1B). Similarly, KLF8 overexpression increased LoVo cell invasion by 415.0% compared with the control cells (Figure 1C). Our results demonstrated that KLF8 overexpression induced EMT and promoted migration and metastasis.

To identify the role of KLF8 in EMT, we first evaluated its response to the most potent EMT inducer, TGF-β1. We showed that TGF-β1 induced KLF8 in a dose-dependent manner, as determined by western blotting. TGF-β1 markedly induced the expression of the EMT marker vimentin (Figure 1D). Blocking TGF-β1 with its neutralizing antibody significantly suppressed endogenous KLF8 expression and TGF-β-induced KLF8 expression in LoVo cells (Figure 1E).

**Figure 2:**
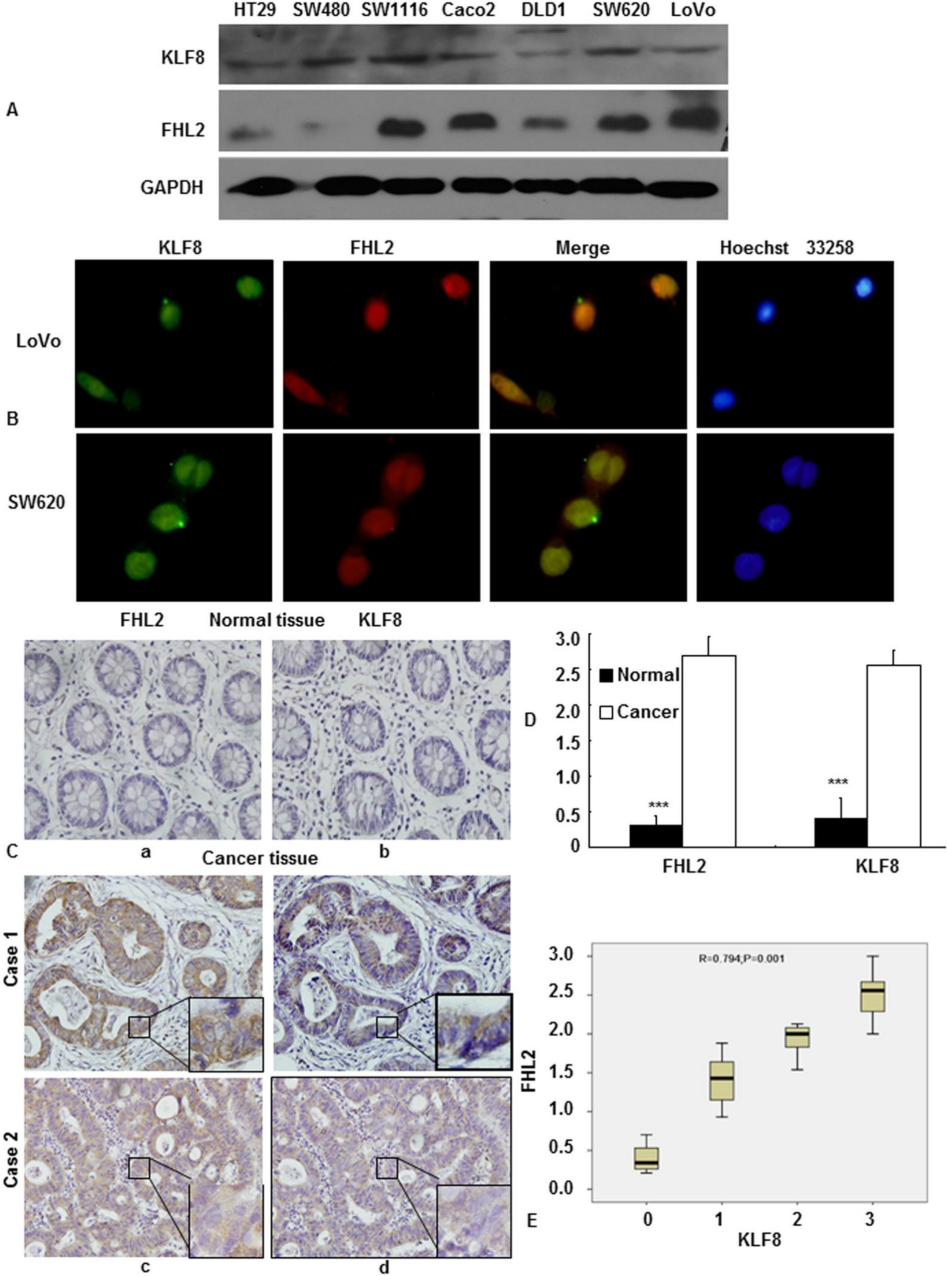
Expression profiles of KLF8 and FHL2 in colon cancer **A.** KLF8 and FHL2 expression was detected in colon cancer cell lines by western blot. GAPDH was used as the internal control. **B.** Double staining of KLF8 and FHL2 in LoVo cells by an indirect immunofluorescence, with the nuclei counterstained by Hoechst 33258 (original magnification, 400×). **C.** FHL2 (a, c) and KLF8 (b, d) expression in normal or cancerous colon tissue specimen was detected by immunohistochemistry. These figures are representative of the patients. **D.** Average scores of the two proteins in normal and cancerous colon tissues. ***, *p* < 0.001 between normal and cancer tissues. **E.** Positive staining for KLF8 and FHL2 was quantified, and their correlation was analyzed using Spearman's correlation method. Scale bars, 20 μm in B and 100 μm in C.

However, pre-transfecting cells with KLF8 siRNA not only inhibited KLF8 expression but also suppressed endogenous and TGF-β-induced vimentin expression (Figure 2F). These results implicated KLF8 as a responsive factor of EMT induction.

### Positive correlation between KLF8 and FHL2 expression in CRC

To elucidate the correlation between KLF8 and FHL2 in cancer cells, we first checked the expression of these proteins in CRC cell lines by western blot. As shown in Figure 2A, KLF8 was expressed at a relatively high level in SW480, SW1116, Caco2 and SW620 cells and at a relatively low level in HT29, DLD1 and LoVo cells. The FHL2 expression pattern was very similar to that of KLF8 in the above 7 cell lines, except for SW480 cells. Secondly, we investigated the cellular distribution of the two proteins. A two-color immunofluorescence assay showed that the endogenous KLF8 and FHL2 proteins localized to both the nucleus and the cytoplasm of LoVo cells and SW620 cells (Figure 2B). A merged signal indicates the co-localization of the two proteins.

To validate our findings *in vivo*, we detected KLF8 and FHL2 expression in 13 pairs of adjacent normal colon mucosal tissues and cancer tissues by immunohistochemistry (IHC), as described above. We showed that both proteins were highly expressed by cancer cells, whereas their expressions in normal tissues were absent or extremely low (Figure 2C). Observations using serial sections showed that KLF8 and FHL2 were distributed in both the nucleus and cytoplasm of the same cancer cells (Figure 2C).

Semiquantitative scoring of the two proteins showed that the expression of both proteins in cancerous tissues was significantly higher than that of adjacent normal colon tissues (Figure 2D), and Spearman's correlation analysis showed a positive correlation between KLF8 and FHL2 expression (correlation coefficient *r* = 0.794, *p* < 0.01, Figure 2E).

### FHL2 is a direct target for transcriptional activation by KLF8

**Figure 3:**
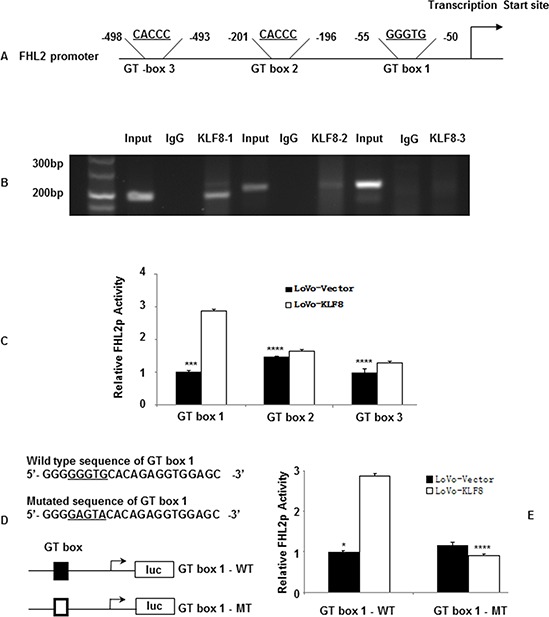
KLF8 directly binds to the promoter of FHL2 **A.** The luciferase (Luc) reporter constructs contained the FHL2 promoter with three potential KLF8 binding sites upstream of a luciferase gene. **B.** Chromatin immunoprecipitation (ChIP) was performed using an anti-KLF8 antibody or control IgG. The FHL2 promoter region to which KLF8 binds was significantly enriched after immunoprecipitation by an anti-KLF8 antibody. PCR products following ChIP were run on an ethidium-stained gel. **C.** KLF8 activates the FHL2 promoter (FHL2p) after co-transfection in LoVo cells. Luciferase activity was measured 48 h after transfection. Luciferase activity is expressed as the ratio of the promoter reporter activity to the control vector luciferase activity. RLU, relative luciferase units. ***, *p* < 0.001; ****, *p* > 0.05 **D.** Primer sequences used for site-directed mutations. A 136-bp (containing GT-box 1) FHL2 promoter fragment was cloned into pGL3 basic to generate pLuc55-wt. Site-directed mutagenesis of GT-box 1 in pLuc55 was performed to generate pLuc55-mt. The locations of GT-box 1 are labeled in bold, and the mutated nucleotides were underlined. wt, wildtype and mt, mutant. Diagram showing the GT-box 1 status in the two constructs. ■, wildtype GT-box 1 and □, mutated GT-box 1. **E.** Transcriptional activities of reporters in the transient transfections. Dual luciferase assay were performed, and the results are expressed as fold induction of RLU. ***, *p* < 0.001; ****, *p* > 0.05.

We next assessed whether KLF8 proteins could directly bind to the FHL2 promoter. We scanned the promoter region (<500 bp) of human FHL2 for the GT-box consensus sequence and found three potential binding sites: −50 to −55 (GT-box 1), −196 to −201 (GT-box 2) and −493 to −498 (GT-box 3) from the transcription initiation site (Figure 3A). To determine whether KLF8 binds to the FHL2 promoter region *in vivo*, we performed ChIP analysis using an anti-KLF8 antibody in LoVo cells. PCR amplification (Figure 3B) showed that a band of 195 bp, containing the first possible binding (−55 to −50) site, was immunoprecipitated. Normal rabbit IgG was used as the negative control. No bands were evident in the immunoprecipitates for the other two possible binding sites (GT-box 2 and/or GT-box 3) or for the control IgG.

We then cloned the promoter regions GT-box 1, GT-box 2 and GT-box 3 of human FHL2 upstream of a luciferase gene in a reporter plasmid. Transient transfections were performed to investigate whether the FHL2 promoter containing only GT-box 1, the only GT-box 1 belonging to the FHL2 promoter, was activated by KLF8 overexpression. The luciferase activity increased by 2.83 ± 0.16 fold compared with that of the vector (*p* < 0.001). However, the reporters that contained GT-box 2 and/or GT-box 3 of FHL2 showed slightly higher or lower luciferase activity compared with that of the vectors (*p* > 0.05; Figure 3C). We next mutated two nucleotides of the identified GT-box 1 binding region (Figure 3D). These mutations greatly reduced the effect of KLF8 on FHL2 promoter activity (Figure 3E). These results suggested that the proximal GT-box at −55 is the main KLF8-binding site in the FHL2 promoter (Figure 3).

### KLF8 promotes FHL2-mediated cell proliferation and morphology in CRC

**Figure 4:**
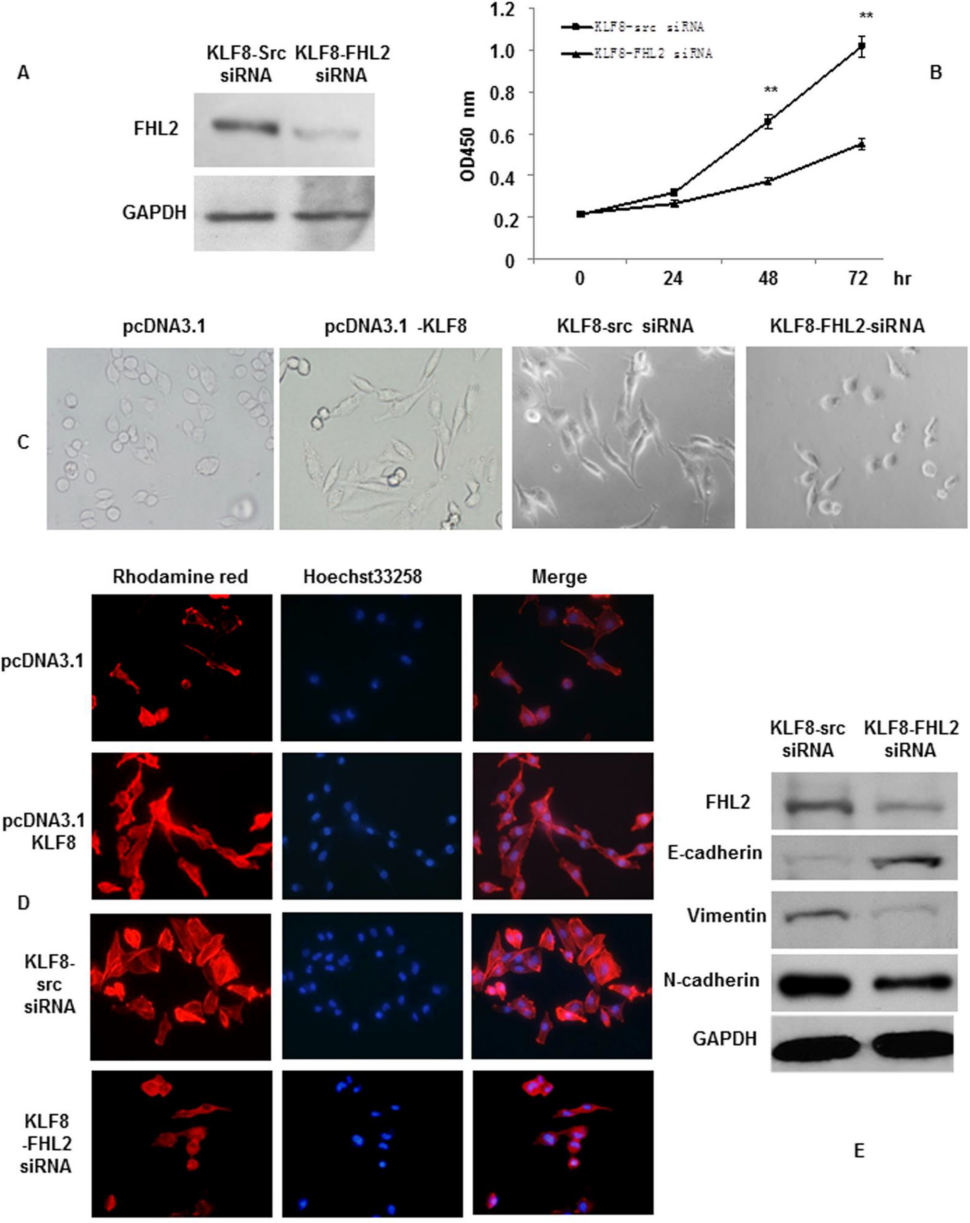
KLF8 promotes FHL2-mediated cell proliferation and morphology in CRC **A.** Stable KLF8 transfectants were transfected with FHL2 siRNA, and FHL2 expression was detected by western blot 48 h later. **B.** LoVo/KLF8-src siRNA and LoVo/KLF8-FHL2 siRNA cells seeded in 96-well tissue culture plates in triplicate and cultured in complete medium for 24, 48 and 72 h were evaluated. Cell proliferation was assessed using the WST-1 assay. **, *p* < 0.01. **C.** Morphology of LoVo/vector, LoVo/KLF8, LoVo/KLF8-src siRNA and LoVo/KLF8-FHL2 siRNA cells, visualized by phase-contrast microscopy. **D.** LoVo cells stained with rhodamine-phallotoxin for 48 h to identify F-actin filaments were visualized under fluorescent microscopy. All experiments were repeated 2 to 3 times with similar findings. **E.** EMT biomarkers, including E-cadherin, N-cadherin, vimentin, and FHL2, were detected by western blot 48 h after transfection. Scale bars represent 20 μm in C and D.

We previously showed that knocking down KLF8 inhibited CRC cell proliferation (Shi X, submitted). To investigate whether the mechanism of KLF8 regulating FHL2 plays a role in cell proliferation, we downregulated FHL2 in KLF8-overexpressing cells using siRNA and confirmed this effect by western blot (Figure 4A). FHL2 downregulation decreased KLF8-mediated proliferation of LoVo cells, as shown using a WST-1 assay. The OD 450 s of KLF8 + FHL2 siRNA cells were 0.2178% ± 0.009%, 0.2646% ± 0.009%, 0.3703 ± 0.01% and 0.5504% ± 0.027% after culturing for 0, 24, 48 and 72 h, respectively, whereas those of KLF8 + src siRNA cells were 0.2122% ± 0.005%, 0.3148% ± 0.012%, 0.6544% ± 0.05% and 1.0136% ± 0.05%, respectively. A significant difference was found between the stable KLF8 + FHL2 siRNA and KLF8 + src siRNA transfectants at 48 and 72 h (*p* < 0.01; Figure 4B).

We then examined the morphologic features of these cells. The stable vector transfectants displayed a round or flat morphology with a short cytoplasmic process. However, the KLF8 transfectants exhibited a spindle-like, fibroblastic morphology, which is one of the main characteristics of EMT. Long or dendritic-like cytoplasmic processes were visible under a phase-contrast microscope. FHL2 knockdown in KLF8-overexpressing cells led to EMT reversion (Figure 4C).

To further characterize KLF8, we stained F-actin using phalloidin staining. Compared with the empty vector-expressing cells, the stable high expression of LoVo of KLF8 cell was present throughout the cytoplasm and at the rim zone of the protrusion. Moreover, filopodia and lamellipodia were identified as dynamic cellular features on the cell membrane surfaces that require actin polymerization and are involved in cancer cell invasion and metastasis (Figure 4D). FHL2 knockdown in KLF8-overexpressing cells led to the mesenchymal to epithelial transition (MET) process, which is the reverse of the EMT process.

Our previous results showed that FHL2 induced tumor cell EMT and maintained the invasive potential of cancer cells [23]. However, the role of FHL2 in KLF8-induced EMT in colorectal cells remains elusive. SiRNA-mediated repression of FHL2 was performed in LoVo-KLF8 cells and resulted in decreased vimentin expression as well as in the conversion from mesenchymal marker (vimentin and N-cadherin) to epithelial marker (E-cadherin) expression compared with the control (KLF8-src siRNA) cells (Figure 4E). These results might indicate that FHL2 and KLF8 regulate cell growth, migration and invasion.

### KLF8 is required for FHL2-mediated EMT and metastatic phenotypes

**Figure 5:**
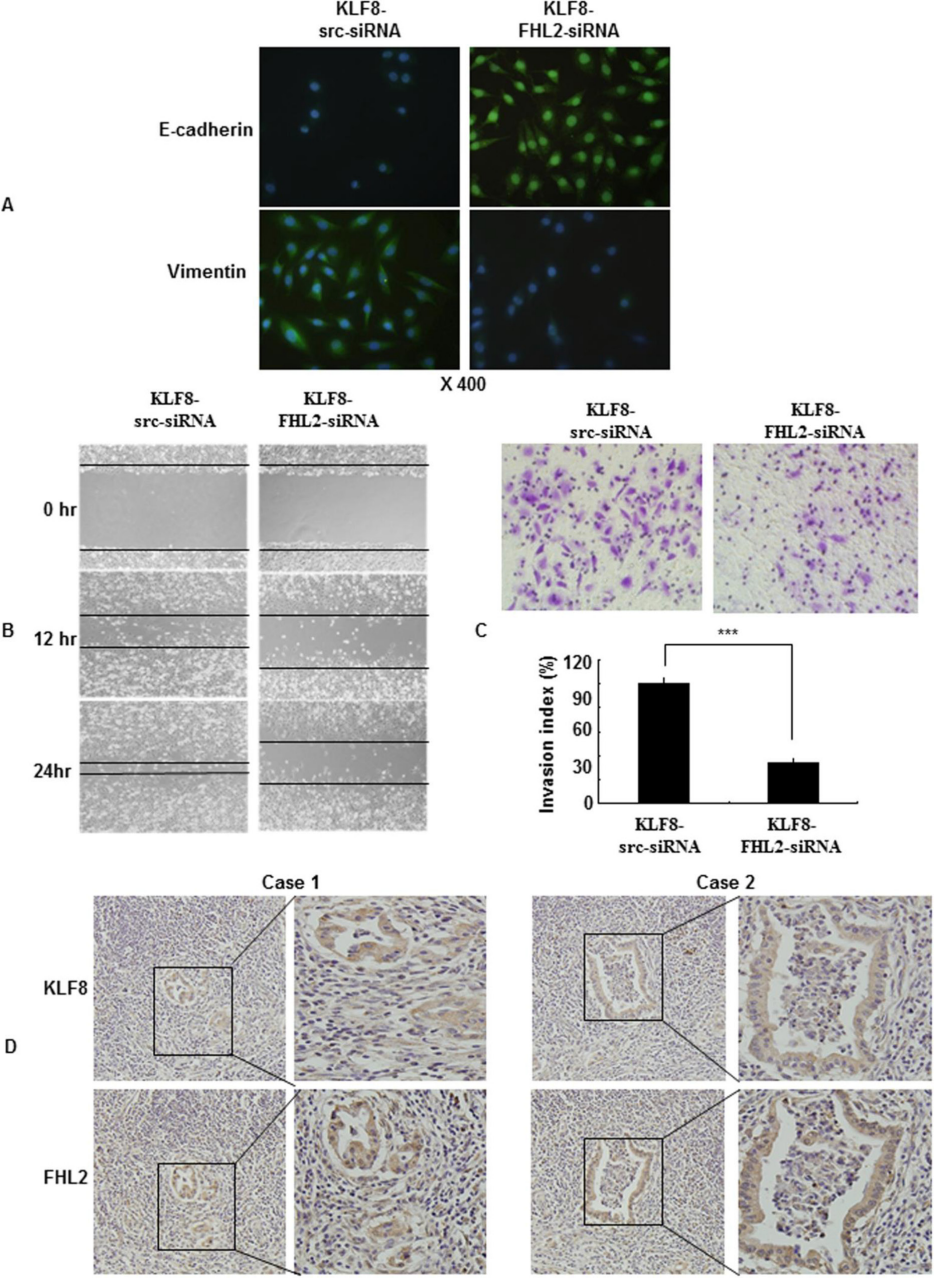
KLF8 is required for FHL2-mediated EMT and metastatic phenotypes **A.** Immunofluorescence and microscopic visualization of E-cadherin (blue) and vimentin (green) staining in KLF8 src siRNA and KLF8-FHL2-siRNA cells. **B.** For the wound-healing experiments, cells were analyzed with live-cell microscopy. Original magnification, 10×. **C.** LoVo stable KLF8 transfectants were transfected with FHL2 siRNA 48 h later, and the invasive ability of the cells decreased. ***, *p* < 0.001. The experiments were repeated at least three times. **D.** Representative IHC images are shown for FHL2 and KLF8 expression in lymph node metastatic cancer tissues. Scale bars, 20 μm in A and 100 μm in D.

Immunofluorescence staining of E-cadherin and vimentin, visualized by microscopy, confirmed the EMT-associated shift in marker expression (Figure 5A). Next, we performed a wound-healing assay. Knockdown of FHL2 in KLF8-overexpressing cells led to a decreased the migratory potential of KLF8-overexpressing cells *in vitro* (Figure 5B). Similarly, FHL2 downregulation in KLF8-overexpressing cells led to a decreased invasion potential of KLF8-overexpressing cells by 34.5% (Figure 5C).

### Knockdown of FHL2 decreased the migratory and invasive potential of KLF8- overexpressing cells

To validate our findings *in vivo*, we detected FHL2 and KLF8 in serial sections of lymph node metastatic cancer tissues as exemplified in two patients. We showed that FHL2 and KLF8 were expressed at high and intermediate levels, respectively, in the cytoplasm and nucleus of cancer cells (Figure 5D). This result confirmed the positive correlation between FHL2 and KLF8 by immunohistochemistry, which is consistent with previous reports.

Taken together, these results demonstrated the critical role of FHL2 in the induction of EMT and in the migration and invasion phenotypes caused by abnormal KLF8 expression.

### KLF8 promotes FHL2-mediated cell proliferation and metastasis *in vivo*

**Figure 6:**
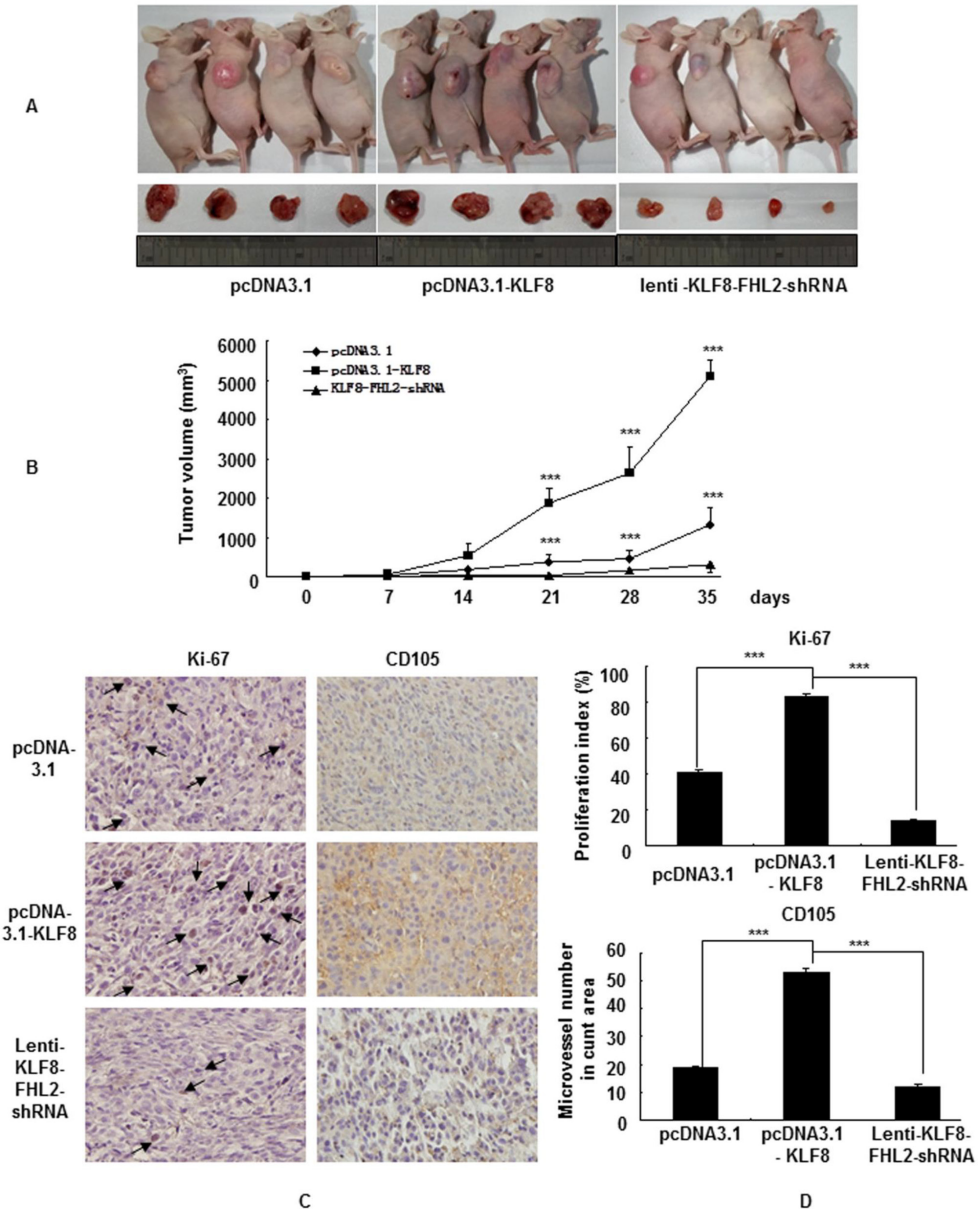
KLF8 promotes FHL2-mediated cell proliferation in CRC *in vivo* **A.** LoVo cells (5 × 10^7^) were injected subcutaneously in the right flanks of nude mice. One week after tumor cell injection, equal amounts of vector, KLF8 or KLF8-FHL2-shRNA cells were directly injected into the tumors at three different positions. Images shown were captured on day 35 after injection. **B.** Tumor size was measured weekly after tumor cell inoculation in each group. ***, *p* < 0.001, vector vs. KLF8 and KLF8 vs. KLF8-FHL2-shRNA, respectively. **C.** FHL2 knockdown significantly inhibited KLF8-induced proliferation (Ki-67, ***, *p* < 0.001, vector vs. KLF8 and KLF8 or KLF8-FHL2-shRNA, respectively), and a considerable decrease of tumor vessel density (CD105, ***, *p* < 0.001, vector vs. KLF8 and KLF8 or KLF8-FHL2-shRNA) was observed by immunohistochemistry. Scale bars represent 100 μm.

To validate our findings *in vivo*, we inoculated vector, stable KLF8-transfectant and Lenti-KLF8-FHL2-shRNA cells into BALB/c-*nu*/*nu* mice, as shown in Figure 6A. The tumor volumes of the KLF8-overexpressing cells were markedly greater than those of the vector-expressing cells. KLF8 overexpression progressed from a pronounced increase in vector cells at day 21 to a 4-fold increase in the cancer area 35 days after injection (Figure 6B). In contrast, tumors derived from FHL2 downregulation in KLF8-overexpressing cells were markedly smaller than those of the vector-treated mice at 3 to 5 weeks (Figure 6B). FHL2 knockdown inhibited the proliferation of KLF8-overexpressing LoVo CRC cells *in vivo*.

We next examined the expression of cell proliferation (Ki-67) and angiogenesis (CD105) markers at the protein levels in the xenograft tumors. Representative images of the tumors, stained by immunohistochemistry, are shown in Figure 6C. The KLF8 stable-transfectant group showed significantly increased proliferation rates and tumor vessel density than those in the vector group, whereas knockdown of FHL2 inhibited the growth rate and tumor vessel density in the KLF8-overexpressing group.

**Figure 7:**
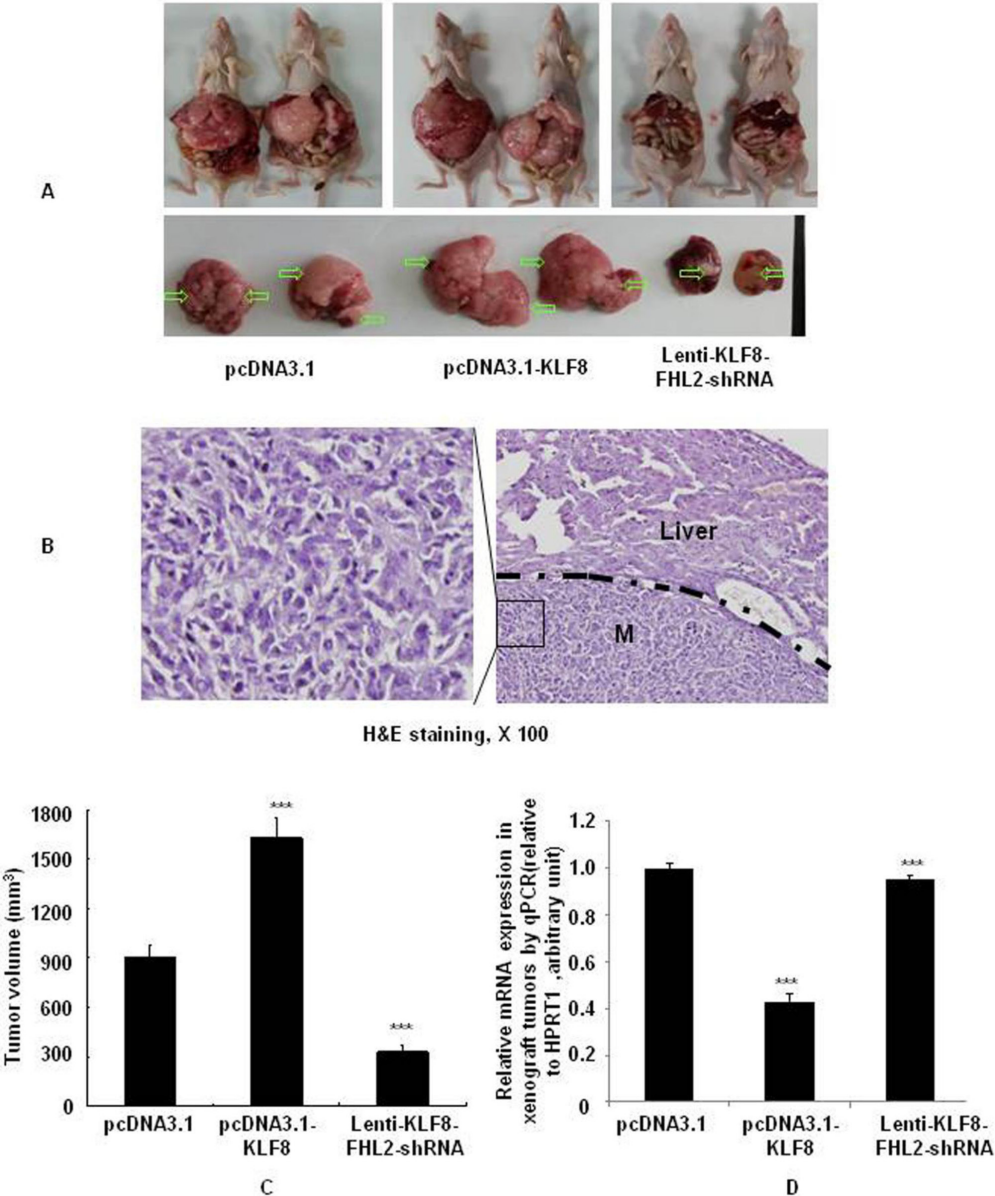
KLF8 is required for FHL2-mediated EMT and liver metastasis in orthotopic tumors after intrasplenic injection **A.** Necropsy images of mice with orthotopically implanted CRC, with metastatic loci marked by arrows. **B.** The mice were sacrificed, and metastatic cancer tissues were stained with H&E. Scale bars, 100 μm. **C.** Tumor volumes (mean ± standard error; ***, *p* < 0.001, vector vs. KLF8; KLF8 vs. KLF8-FHL2-siRNA) in mice measured on the last day of the experiment, at autopsy (*n* = 2). **D.** The expression of E-cadherin in tumors derived from LoVo cells was determined by qPCR. ***, *p* < 0.001, vector vs. KLF8; KLF8 vs. KLF8-FHL2-siRNA.

To investigate whether FHL2 is required for KLF8-induced metastatic phenotypes, shRNA-mediated repression of FHL2 was performed in KLF8-overexpression in LoVo after orthotopic implantation assays [35] (Figure 7A). The presence of CRC metastasis in the liver was confirmed by histological analysis (Figure 7B). The tumor volumes of the KLF8-overexpressing group were significantly increased compared with those of the vector group (*p* < 0.001 vs. vector), whereas the tumor volumes after FHL2 knockdown were greatly decreased compared with those in the KLF8-overexpressing group on day 23 (Figure 7C). These results demonstrated that FHL2 downregulation in KLF8-overexpressing cells inhibited CRC cell metastasis to the liver.

To further demonstrate whether KLF8 is required for FHL2-mediated EMT, repression of FHL2 in KLF8-overexpressing cells was performed at the mRNA level in orthotopic xenograft tumors. KLF8 overexpression resulted in a significant loss of the epithelial marker E-cadherin, whereas FHL2 downregulation in KLF8-overexpressing cells increased E-cadherin (Figure 7D). Thus, KLF8 is suggested to play a critical role in the induction of EMT via FHL2 expression.

Taken together, these results clearly indicated that the KLF8-FHL2 axis plays an important role in CRC development and metastasis.

## DISCUSSION

In this report, we identified the KLF8-mediated regulation of FHL2 as a novel mechanism for the promotion of human CRC cell proliferation, invasion and metastasis. First, we showed a strong correlation between the expression of KLF8 and FHL2 in CRC cell lines. Second, we determined that KLF8 promoted FHL2 transcription and activity. Third, our data showed that KLF8 had a critical role in FHL2-mediated tumor growth, EMT and metastatic phenotypes *in vitro* and *in vivo*. These findings suggested that the cooperative relationship between FHL2 and KLF8 plays a pivotal role in CRC progression and metastasis.

KLF8 is a GT-box binding dual-transcription factor, and KLF8 overexpression has been noted in a variety of aggressive human carcinomas [16, 19]. Interestingly, several studies on KLF8 have indicated its involvement in the early steps of metastasis. For example, KLF8 was shown to stimulate the invasion of breast cancer cells through the induction of the matrix metalloproteinase gene MMP-9 [17]. Our results showed that KLF8 overexpression induced EMT and promoted migration and metastasis in CRC cells. These findings implied that aberrant upregulation of KLF8 might be an important mechanism underlying cancer metastasis.

TGF-β1 has also been shown to promote tumor progression and metastasis in established cancers, in part through the induction of EMT [23, 36–38]. This apparent conversion in TGF-β1 function after tumor initiation is known as the “TGF-β1 paradox.” It is important to understand the downstream effectors of the TGF-β1 signaling pathway and to identify the “molecular switch” or “switches” mediating the “TGF-β1 paradox.” TGF-β1 is considered a major driver of the EMT in tumors. While investigating the mechanistic interactions between TGF-β1 and KLF8 signaling during induction of EMT in CRC, we found that induction of EMT in LoVo cells by TGF-β1 was associated with a significant increase in KLF8 expression. When KLF8 expression was silenced, TGF-β1 induction of the EMT phenotype was abrogated, and KLF8 expression was also decreased. We suggest that KLF8 might act as a potent inducer of EMT in CRC cells.

FHL2 is a member of the FHL protein family [20]. The role of FHL2 in cancer is particularly intriguing because FHL2 binds to different proteins [24–26]. In rhabdomyosarcoma cells, FHL2 overexpression induced apoptosis [20]. In melanoma cells, however, the synergistic overexpression of SKI and FHL2 enhanced cell growth [28]. Thus, it appears that FHL2 can regulate tumorigenesis in multiple human cancers [23, 27]. However, the roles of KLF8 and FHL2 in CRC are unknown. In this study, we showed that the distribution pattern of FHL2 was highly congruent with that of KLF8 and that FHL2 protein expression was highly correlated with KLF8 expression by immunohistochemistry and immunofluorescence. Moreover, FHL2 knockdown resulted in a marked blockage of KLF8 expression *in vitro*. Therefore, we speculated that KLF8 and FHL2 might play roles in CRC progression.

The Sp1/KLF family members are related in that they bind GC-box and CACCC-box sequences in gene promoter regions [17]. We previously demonstrated that Sp1 stimulated FHL2 expression in GI cancer cells through interaction with its binding element located at the promoter of the FHL2 gene [3]. However, whether FHL2 is involved with KLF8 function remains unclear. In the present study, we identified that FHL2 is a direct transcriptional target of KLF8, evidenced by KLF8 directly binding to and activating the FHL2 promoter. This result provides a mechanism by which KLF8 induced FHL2-mediated EMT and potentially contributed to CRC growth and metastasis. The conclusion is based on the following observations. First, we showed that KLF8 directly binds to the FHL2 promoter by ChIP and luciferase reporter assays. The results from these studies identified the sequence from −55 to −50 bp as GT-box 1; furthermore, mutation of GT-box 1 resulted in blockage of KLF8 transcriptional activity. Second, we further investigated whether KLF8 contributed to FHL2-induced EMT. By silencing FHL2 expression in LoVo cells transfected with a KLF8-expressing plasmid, we found that the EMT phenotype was reversed. Third, FHL2 knockdown inhibited the EMT and metastatic potential induced by KLF8 overexpression in CRC *in vivo*. Together, these results strongly suggested that the FHL2-induced EMT and metastasis were mediated through KLF8.

In summary, we identified FHL2 as a novel target for transcriptional activation by KLF8 and demonstrated that the regulation of FHL2 by KLF8 is critical for human CRC cell growth and invasion. We have also shown that KLF8 expression was important for human CRC EMT and metastasis and played an important role in CRC cell progression and aggression. Therefore, KLF8 might represent a novel favorable target for intervention against CRC cell proliferation and metastasis.

## MATERIALS AND METHODS

### Reagents and antibodies

Recombinant human TGF-β1 (240-B) and monoclonal anti-TGF-β1 antibody were purchased from R&D Systems (Minneapolis, MN, USA). Mouse anti-human FHL2, used for western blotting, was purchased from MBL International Incorporation (Woburn, Japan). Rabbit anti-human KLF8 antibody, used for western blotting and IHC, was purchased from Aviva Systems Biology (San Diego, CA, USA). Prediluted rabbit anti-human vimentin, N-cadherin and E-cadherin, KLF8 and mouse anti-human glyceraldehyde-3- phosphate dehydrogenase (GAPDH) antibodies, used for western blotting and/or immunohistochemistry, were purchased from Abcam (Cambridge, UK). Bovine anti-mouse IgG-TR and goat anti-rabbit IgG-FITC antibodies purchased from Santa Cruz (Santa Cruz, CA, USA). Goat anti-rabbit immunoglobulins/HRP, rabbit anti-mouse immunoglobulins/HRP, and normal mouse IgG antibodies were purchased from Dako (Carpinteria, CA, USA).

The human colon cancer cell lines HT29, SW480, SW1116, Caco2, DLD1, SW620 and LoVo were obtained from American Type Culture Collection (ATCC, Rockville, MD, USA) and cultured as previously described [16].

### Immunohistochemistry of human CRC cancer samples

The Ethics Committee of the Southern Medical University of China approved our experimental protocols. Thirteen pairs of surgically resected colon cancer tissues and adjacent normal tissues were collected from both primary and metastatic (lymph node) cancer sites (Department of Pathology, Nanfang Hospital, First Affiliated Hospital of Southern Medical University, China). The expression of FHL2 and KLF8 was visualized as described previously. The tissues in which more than 10% of cancer cells were positively stained were considered positive. For the quantitative analysis, the ratio of positively stained cells to all of the tumor cells in five random areas at 200-fold magnification was recorded. Scoring of the tissue slides was independently performed by two investigators; the percentage of positive cells and the intensity of staining were scored from 0 to 3 as follows: 0, less than 10% of cells stained; 1, 10–50% of cells stained; 2, 50–75% of cells stained; and 3, more than 75% of cells stained.

### Constructs and establishment of stable transfectants

The KLF8 open reading frame and 3′-untranslated region (774 bp) were cloned into pcDNA3.1(+) in a previous study [19]. To establish stable cell lines, cells transfected with empty pcDNA 3.1 vector and pcDNA3.1-KLF8 were passaged 1:15 (vol/vol) and cultured in RPMI 1640 medium (Invitrogen, Carlsbad, CA) supplemented with 800 μg/ml geneticin (G418, Calbiochem, Darmstadt, Germany Canada) for 4 weeks.

### Cell migration and invasion assays

Cell migration was assessed using a wound-healing assay. Cells plated in six-well plates at 90–95% confluence were wounded with a pipette tip at time 0. The speed of wound closure was monitored, and the cells were imaged at different time points using a phase-contrast microscope. The media were changed to remove cell debris, and the cells were cultured in the presence of 10 μg/ml mitomycin C to inhibit cell proliferation. Images were captured 24 h later.

Cell invasion was assessed using a Matrigel invasion chamber (BD Biosciences), following the protocol provided by the manufacturer. After incubation for 48 hours, the noninvasive cells were removed with a cotton swab. Invasive cells were stained with 0.2% crystal violet and counted under a microscope. The average number of cells in five fields per membrane was counted in triplicate inserts. The invasion index was expressed as the percentage of test cells to that of treatments cells or control.

### siRNA transfection

The siRNA duplexes consisted of 21 base pairs with a 2-base deoxynucleotide overhang (Proligo, Singapore). The siRNA sequences were as follows: KLF8 sense strand, ((NM_007250), 5′-CGAUAUGGAUAAACUCAUAdTdT-3′; and FHL2 sense strand, (NM_201557), 5′-CGAAU CUCUCUUUG GCAAGdTdT-3′. The scrambled (src) siRNA (5′-TTC TCCGAACGTGTCACGT-3′), which does not target any gene, was used as the negative control. The siRNA transfection was performed using Lipofectamine 2000 (Invitrogen, USA), according to the manufacturer's instructions.

### Construction and transfection of lentiviral vectors

To further investigate the effects of siRNA-induced down-regulation of FHL2 in KLF8-overexpressed cells on tumor growth *in vivo*, an FHL2-RNAi lentiviral vector (pGCSIL-FHL2shRNA) was constructed (Shanghai GeneChem Co, Ltd, Shanghai, China) [19]. Double-stranded oligonucleotides encoding human FHL2-vshRNA (CCGGCCGAATCTCTCTTTGGCAAGTCAAGAGCT TGCCAAAGAGAGATTCGTTTTTG) were inserted into the short hairpin RNA (shRNA) expression vector pGCSIL (Shanghai GeneChem Co., Ltd.), and the identities of the clones were verified by sequencing.

A recombinant lentiviral vector was produced by co-transfecting HEK293T cells with the lentiviral expression vector and the packaging plasmid mix using Lipofectamine 2000, according to the manufacturer's instructions.

The titer of the purified virus was determined in 293T cells using the serial dilution method.

For lentiviral transduction, LoVo cells were subcultured at 1 × 10^5^ cells/well in 6-well culture plates and were then transduced with FHL2-siRNA-expressing (FHL2 siRNA) or src-siRNA-expressing lentivirus at a 50 multiplicity of infection. The cells were harvested 72 h after infection, and the transduction efficiency was evaluated by counting the percentage of GFP-positive cells.

### Western blot assays

For western blot analysis, cells were harvested and lysed in lysis buffer. A total of 30 μg of protein lysates were separated by SDS-PAGE and transferred onto a PVDF membrane. Primary antibodies were diluted according to the company's recommendation. Proteins were visualized using the enhanced chemiluminescence detection system.

### Reverse transcription PCR (RT-PCR) and quantitative real-time reverse transcription PCR (qRT-PCR)

Total RNA extraction and reverse transcription were performed as previously described [31]. Quantitative real-time RT-PCR was performed using Applied Biosystems Sequence Detection System 7900 (ABI Prism 7900HT, Applied Biosystems Company, Foster City) with 10 ml of a mixture comprising Power SYBR GREEN PCR Master Mix Applied Biosystems Company, 500 nmol of each primer and 300 ng of complementary DNA template. The reactions were performed with an initial denaturation step of 95°C for 5 min, followed by 60 cycles of 20 s at 94°C, 20 s at 55°C and 40 s at 72°C. A final extension at 72°C for 5 min was included before a temperature ramp from 72°C to 95°C at 0.1°C/s with continuous fluorescent acquisition. Each complementary DNA sample was run in duplicate for each real-time PCR assay, and the average relative fold messenger RNA levels were determined using the 2^−ΔΔCt^ method, with HPRT1 detected as the internal control [39, 40].

### Promoter reporter and dual luciferase assays

First, 55-bp, 201-bp and 498-bp fragments of the FHL2 promoter upstream of the transcription start site were cloned into the pGL3basic vector. The pLuc55 mutant construct was created by site-directed mutagenesis of the FHL2 promoter vector (Stratagene, La Jolla, CA, USA). The primer sequences are listed in Supplementary Table S1.

For the luciferase assay, the cells were transiently transfected with the various pLuc constructs with Lipofectamine 2000, as previously reported [23]. The firefly and Renilla luciferase activities were measured using the Dual-Luciferase reporter system (Promega) with a model TD-20/20 Luminometer (EG&G, Berthold, Australia). The firefly luciferase activity value was normalized to the Renilla activity value. The transcriptional activity at the promoter was presented as the fold induction of relative luciferase units (RLUs) compared with the basic pGL3 vector control. The RLU was the value of the firefly luciferase unit divided by the value of the Renilla luciferase unit.

### Chromatin immunoprecipitation (ChIP) assays

ChIP assays were performed according to the protocol provided by the ChIP assay kit (Upstate Cell Signaling Solutions, Lake Placid, NY, USA). The lysates were incubated either with antibodies specific for KLF8 or with normal mouse IgG. The PCR reaction generated 195-bp, 225-bp and 228-bp products from the FHL2 proximal promoter containing GT-box 1 (−56 to −51 bp), GT-box 2 (−202 to −197 bp) and GT-box 3 (−499 to −494 bp), respectively. The primers and antibodies used in the ChIP assays are listed in Supplementary Table S1.

### Immunofluorescence

Cells grown on glass coverslips were fixed with 4% paraformaldehyde, and non-specific binding was blocked. The coverslips were probed with primary antibodies, followed by Texas red (TR)- or FITC-conjugated secondary antibodies. The nuclei were counterstained with 1 μg/ml Hoechst 33258. After mounting, the coverslips were visualized under a Zeiss Axioskop fluorescence microscope.

### WST-1 cell proliferation assay

Scr-siRNA and KLF8-siRNA cells were seeded into 96-well plates at 0.5–1 × 10^4^ cells/well and cultured in RPMI 1640 for 24, 48 and 72 h. Then, 10 μl of the WST-1 reagent (Roche Diagnostics, Mannheim, Germany) was added to each well, and the plates were incubated for 4 h at 37°C. The amount of formazan was quantified using an ELISA reader at an absorbance of 450 nm.

### *In vivo* tumor growth assay

Tumor growth was evaluated in a nude mouse xenograft model. LoVo cells (5 × 10^6^), in 0.1 ml of RPMI, were inoculated subcutaneously into the right flanks of 5–6-week-old female BALB/c-nu/nu mice (Laboratory Animal Unit, Southern Medical University, China), and the resulting tumor sizes were measured weekly. Institutional guidelines were followed for the handling of the animals. The mice were maintained under sterile conditions. The tumor volumes were calculated as follows: total tumor volume (mm^3^) = L × W^2^/2, where L is the length, and W is the width. On day 35 after inoculation, the mice were sacrificed, and the tumors were dissected and weighed. Immunohistochemistry analysis was performed using anti-Ki-67, MMP-9 and anti-CD105 antibodies.

### *In vivo* metastasis assays

The mice were anesthetized with isoflurane. For the orthotopic tumor implantation assays, pcDNA3.1-, pcDNA3.1-KLF8- or lenti-KLF8-FHL2-siRNA-expressing cells (1 × 10^6^ in 0.1 ml of PBS) were inoculated into the dorsal subcostal incision to expose the spleen. The volume (50 μL) of tumor cell suspension was slowly injected into the spleen using a 25-gauge needle. After 23 days, the mice were euthanized. The weight and volume of the livers were measured for each mouse. Metastatic tissues were microscopically examined with H&E staining.

### Statistical analyses

The results obtained from duplicated q-RT-PCR assays and triplicated luciferase experiments were expressed as the mean ± SD. The significance of the patient specimen data was determined using the Pearson correlation coefficient. The significance of the *in vitro* and *in vivo* data was determined using Student's *t* test (two-tailed). *P* values less than 0.05 were considered significant.

## SUPPLEMENTARY MATERIALS AND METHODS TABLE



## Abbreviations

EMT: epithelial-to-mesenchymal transition
FHL2: four-and-a-half LIM-only protein 2
RLU: relative luciferase unit
ATCC: american type culture collection
ChIP: chromatin immunoprecipitation.
