# Supplementary material for: KLF8 promotes tumorigenesis, invasion and metastasis of colorectal cancer cells by transcriptional activation of FHL2
Source: Oncotarget. 2015 Jul 15;6(28):25402–17. doi: 10.18632/oncotarget.4517 (PMC4694840; doi:10.18632/oncotarget.4517)
Supplement: Supplementary file 1 [file oncotarget-06-25402-s001.pdf]

SUPPLEMENTARY MATERIALS AND METHODS

Reagents, cell lines and tissue specimens

Recombinant human TGF-β1 (240-B) and monoclonal anti-TGF-β1 antibody were purchased from R&D Systems (Minneapolis, MN, USA). Mouse anti-human FHL2, used for western blotting, was purchased from MBL International Incorporation (Woburn, Japan). Rabbit anti-human KLF8 antibody, used for western blotting and IHC, was purchased from Aviva Systems Biology (San Diego, CA, USA). Prediluted rabbit anti-human vimentin, N-cadherin and E-cadherin, KLF8 and mouse anti-human glyceraldehyde-3- phosphate dehydrogenase (GAPDH) antibodies, used for western blotting and/or immunohistochemistry, were purchased from Abcam (Cambridge, UK). Bovine anti-mouse IgG-TR and goat anti-rabbit IgG-FITC antibodies purchased from Santa Cruz (Santa Cruz, CA, USA). Goat anti-rabbit immunoglobulins/HRP, rabbit anti-mouse immunoglobulins/HRP, and normal mouse IgG antibodies were purchased from Dako (Carpinteria, CA, USA).

The human colon cancer cell lines HT29, SW480, SW1116, Caco2, DLD1, SW620 and LoVo were obtained from American Type Culture Collection (ATCC, Rockville, MD, USA) and cultured as previously described [14].

SUPPLEMENTARY TABLE

Supplementary Table S1: List of the oligonucleotides primers used for amplification and mutation

Experiment	Name	Position or orientation	Sequence (5′-3′)
qRT-PCR	E-cadherin	550 ~ 570	F: 5′- TGCCCAGAAAATGAAAAAGG -3′
		730 ~ 751	R: 5′- GTGTATGTGGCAATGCGTTC -3′
	HPRT1	147 ~ 166	F: 5′- TGCTCGAGATGTGATGAAGG -3′
		318 ~ 338	R: 5′- TCCCCTGTTGACTGGTCATT -3′
Luciferase constructs	R	–79 ~ –58	TCCGCTCGAG CGAGGCCTCATATTTTCCAG (Xho I)
	pLuc55 (GT-box 1)	–58 ~ –36	F: 5′ - GGGGTACC GGGGGGTGCACAGAGGTGGAGC (Kpn I) -3′
	pLuc201 (GT-box 2)	–210 ~ –190	F: 5′- GGGGTACC ACATCCTCTCACCCAAGCAA (Kpn I) -3′
	pLuc498 (GT-box 3)	–500 ~ –480	F: 5′- GGGGTACC ATGGGTGAGTGCCTTTTCAG (Kpn I) -3′
Site-directed mutagenesis	pLuc55-MT	–58 ~ –36	F:CCTAATCTGGGGAGTACACAGAGGTGGAGCTGAGCAGCC
			R:GGCTGCTCAGCTCCACCTCTGTGTACTCCCCAGATTAGG
ChIP	ChIP 1	L: –145 ~ –125	ATCCACATTTCCCAGCTCAC
		R: 30 ~ 50	AGGGTGGCTAAACTGGGAAG
	ChIP 2	L: –306 ~ –286	GGAGGATAGGGAGGGAGAGA
		R: –102 ~ –83	CCCAGGAGTGAAAGGACACT
	ChIP 3	L: –620 ~ –600	CAGCTGTATCTCTGCCCACA
		R: –413 ~ –392	TTAAAGCCATATGCTGGGAGA
